# Can Dietary Actives Affect miRNAs and Alter the Course or Prevent Colorectal Cancer?

**DOI:** 10.3390/ijms241210142

**Published:** 2023-06-14

**Authors:** Monika Prendecka-Wróbel, Dominika Pigoń-Zając, Daria Sondej, Karolina Grzywna, Katarzyna Kamińska, Mariusz Szuta, Teresa Małecka-Massalska

**Affiliations:** 1 Department of Human Physiology of the Chair of Preclinical Sciences, Medical University of Lublin, Radziwiłłowska 11, 20-080 Lublin, Poland; dominika.pigon-zajac@umlub.pl (D.P.-Z.); daria.sondej@wp.pl (D.S.); katarzynaakaminska00@gmail.com (K.K.); teresa.malecka-massalska@umlub.pl (T.M.-M.); 2 Faculty of Medical Sciences in Zabrze, Medical University of Silesia, 40-055 Katowice, Poland; karolina.grzywna5@gmail.com; 3 Chair of Oral Surgery, Jagiellonian University Medical College, 31-155 Kraków, Poland; m.szuta@wp.pl

**Keywords:** miRNA, colon cancer, diet

## Abstract

Colorectal cancer is a diet-related cancer. There is much research into the effects of nutrients on the prevention, modulation, and treatment of colorectal cancer. Researchers are trying to find a correlation between epidemiological observations indicating certain dietary components as the originator in the process of developing colorectal cancer, such as a diet rich in saturated animal fats, and dietary components that could eliminate the impact of harmful elements of the daily nutritional routine, i.e., substances such as polyunsaturated fatty acids, curcumin, or resveratrol. Nevertheless, it is very important to understand the mechanisms underlying how food works on cancer cells. In this case, microRNA (miRNA) seems to be a very significant research target. MiRNAs participate in many biological processes connected to carcinogenesis, progression, and metastasis. However, this is a field with development prospects ahead. In this paper, we review the most significant and well-studied food ingredients and their effects on various miRNAs involved in colorectal cancer.

## 1. Introduction

Cancer is a genetic disease [1]. The process of oncogenesis is initiated by gene mutations [2]. These mutations usually affect the somatic cells of the body but can also occur in the cells of germinal lines, which are responsible for the occurrence of family neoplasms [3]. One mutation is inadequate for the development of neoplastic disease; it is caused by mutations in at least several genes. Several groups of genes responsible for the formation of cancer have been distinguished: proto-oncogenes, suppressor genes, and genes related to apoptosis [4]. Among the factors associated with the formation of neoplasms, miRNA is also mentioned [3]. Modifications of miRNA expression are important in the process of carcinogenesis, including colorectal cancer (CRC); therefore, they become therapeutic targets. Thus, it may be possible to manipulate miRNAs to improve cases of resistance to radio and chemotherapy [5].

After lung and breast cancer, colorectal cancer is currently the third most frequently diagnosed cancer in the world. Annual mortality is around 900,000, making it the fourth deadliest cancer in the world. High morbidity and mortality is associated with factors such as an aging society and unhealthy eating habits, in addition to smoking, a sedentary lifestyle, and obesity. Modern medicine has a wide range of treatments, including surgical and endoscopic methods, radiotherapy, and systemic treatment based on cytostatic drugs, targeted therapy, and immunotherapy. Due to modern treatment, it is possible to extend the survival of patients with colorectal cancer, which in the case of advanced disease is only about 3 years, but more effective therapeutic options are still being sought [6]. The risk of developing colorectal cancer is strongly related to eating habits. Increased cancer risk is associated with excessive consumption of animal fats [7]. A large amount of animal fats is associated with the development of a specific bacterial flora that is responsible for the breakdown of bile salts and leads to the formation of carcinogenic compounds [8]. High-fat meals cause the release of bile acids, which, when passed into the colon by the action of the intestinal microflora, are transformed into secondary bile acids such as deoxycholic acid (DCA), which are de facto toxic compounds for the genome [9]. The research results also show that the use of probiotics contributing to the maintenance of normal intestinal flora reduces the risk of cancer development [10]. Western diets are also characterized by high consumption of red meat, which also increases cancer risk [8]. Increased incidence of colorectal cancer caused by the consumption of red meat may be related to the presence of a large amount of iron in the form of heme [11]. Compounds such as polycyclic aromatic hydrocarbons or heterocyclic amines also exhibit carcinogenic properties. Many of these components are identified in some meat dishes cooked at high temperatures [11]. Some studies also show an increased risk of colorectal cancer in people whose diets were poor in fruit and vegetables [12]. Colcock et al. confirmed that an increased amount of dietary fiber is a factor in reducing the risk of colorectal cancer [13].

Currently, attention is given to microRNAs as being very important in various tumor signaling pathways related to the quality and quantity of individual dietary components. RNAs are short (~22 nucleotides in length), single-stranded, and non-coding acids that regulate post-transcriptional gene expression and are termed microRNAs [14]. The action of microRNA is based on binding with high or low complementarity to the 3′-UTR (3′ untranslated regions of messenger RNAs) regions of untranslated mRNA, resulting in inhibition of this process or degradation [14]. MiRNA molecules take part in the formation of neoplasms from initiation through to promotion and progression, thus affecting the processes of multiplication, differentiation, and metastasis [15].

### 1.1. Biogenesis of miRNA

**Figure 1:**
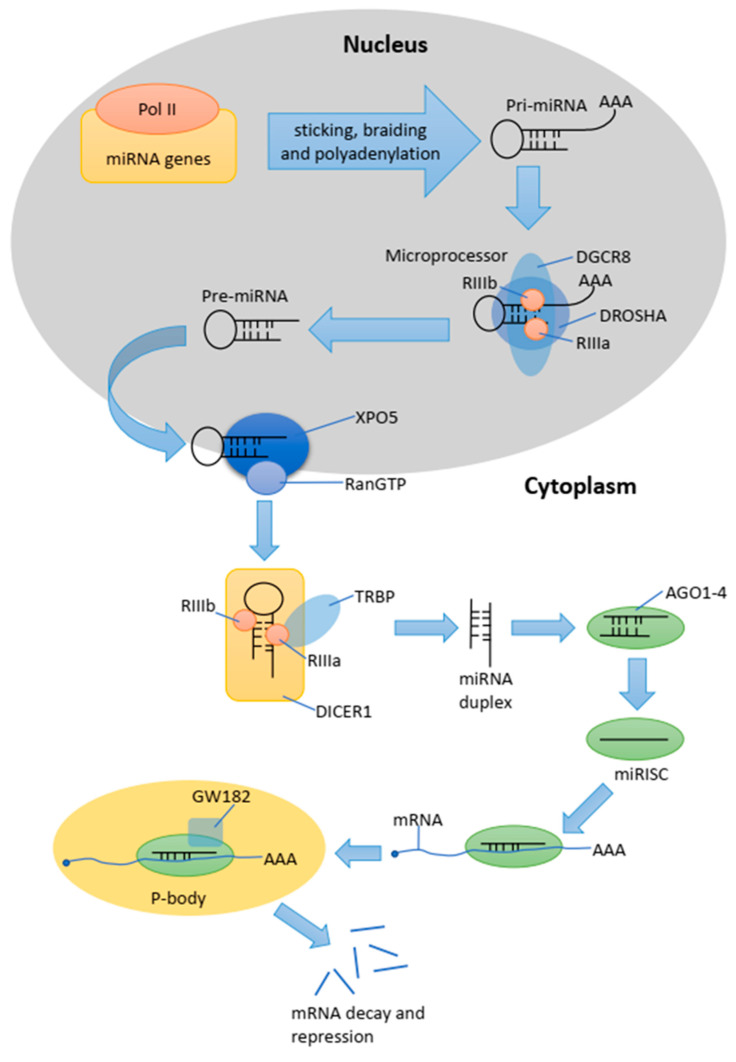
Schematic representation of the miRNA biogenesis pathway.

The cell nucleus is the miRNA synthesis site where the miRNA is transcribed by RNA polymerase II into the primary pri-miRNA transcript. It is shaped like a hairpin and ranges in length from hundreds to thousands of nucleotides [16]. The next steps lead to the formation of regulatory miRNAs. In the initial stage, the so-called pri-miRNA is transformed into smaller units, about 70 nucleotides, which we refer to as pre-miRNA. This is carried out via the microprocessor complex, which consists of DGCR8 (the critical region of the DiGeorge 8 complex) and Drosh RNA polymerase III (DROSHA) [17,18]. In the next step, Exportin 5 (XPO5) transfers the pre-miRNA through special pores in the nuclear membrane into the cytoplasmic space [19,20]. In the cytoplasm, the pre-miRNA is processed by DICER (RNase motif helicase), resulting in a mature miRNA 21–23 nucleotides long [16]. The binding of DICER1 to the end of the pre-miRNA positions its two catalytic RNase III domains such that the asymmetric cleavage of the dsRNA core, near the terminal loop sequence, yields a mature miRNA duplex. DICER1 binds to the transactivation responsive RNA-binding protein (TRBP). TRBP increases the accuracy of pre-miRNA cleavage via DICER1 in a structure-dependent manner and alters the miRNA targeting strand selection by triggering isomiRNA formation [21,22]. TRBP is also a kind of link between DICER1 and Argonaute proteins (AGO1, AGO2, AGO3, andAGO4) to allow the assembly of the miRNA-induced silencing complex (miRISC) [23]. This process takes place in processing bodies (P-bodies), which are the cytoplasmic foci that are induced by mRNA silencing and decay but are not necessarily required for miRNA-mediated gene silencing [24,25]. The mechanism of miRNA biogenesis is presented in Figure 1.

### 1.2. miRNA and Colon Cancer

The discovery of miRNAs completely changed the possibilities and tools in the diagnosis, prognosis, and treatment of a few cancers, including colorectal cancer. By acting on a suppressor gene, for example, microRNAs are involved in all steps leading to CRC development and progression [26,27]. The process of regulating microRNA transcription is complex and diverse, hence the complexity and variability of altered miRNA expression in the CRC. Incorrect miRNA transcription in CRC may therefore be the result of transcription factors that are activated by a variety of oncogenic signaling cascades because of genome amplification/loss, genotoxic stress, or inflammatory stimuli. The miRNA expression of CRC cell lines (in vitro studies) is regulated by DNA methylation as Suzuki et al. reported [28]. Various miRNAs, which include let-7, miR-34, miR-342, miR-345, miR-9,20 miR-129, and miR-137, are frequently hypermethylated in colon tumors, and this is thought to lead to their reduced expression [29,30,31,32]. An example of more global epigenetic regulation in CRC is miR-143, a tumor suppressor microRNA that directly targets DNA methyltransferase 3A (DNMT3A). In this case, loss of miR-143 expression leads to an increase in DNMT3A in CRC tissues [33]. Likewise, loss of miR-342 leads to an increase in DNA methyltransferase 1 (DNMT1), which in turn leads to hypermethylation of several tumor suppressor genes in CRC [34]. The studies of Zhang et al. show that the induction of apoptosis via inhibition of BCL-2 is affected by the upregulation of miR-148a in CRC, while downregulation is associated with an increase in tumor size [35], whereas reduced expression of miR-34a and miR-200c is associated with metastases in CRC [36,37]. Interesting results were obtained by Lujambio et al., who identified that cancer-specific hypermethylation of CpG lets in a promoter change transcribed with miR-148a, miR-34b/c, miR-9, and miR-34a [38]. They found that the above change also influenced the invasion and metastasis of colorectal cancer in combination with IL-6R, ZNF281, MET, zinc finger of the snail family 1 and 2 (SNAI1, SNAI2), and β-catenin (CTNNB1) [36,39,40,41]. Aslam et al. report important proteins involved in key CRC signaling pathways, such as Wnt/β-catenin, phosphatidylinositol 3-kinase (PI3K), KRAS, and tumor protein 53 (p53), which are affected by miRNA expression, indicating a relationship between miRNAs and the development of colorectal cancer [42]. An example is the EGFR/MAPK activation process via downregulating KRAS strongly associated with let-7, miR-18a*, and miR-143 [43,44]. It should be added that miR-126 activates the PI3K pathway [45] while upregulation of miR-21 leads to inhibition of PI3K [46], which in turn leads to increased viability, proliferation, and the beginning of angiogenesis. This type of cancer is particularly difficult to treat, which, in addition to numerous side effects, can cause resistance to chemotherapy. As is known, miRNAs are involved in progression and therefore can be treated as therapeutic targets [47].

Due to the ability of miRNAs to target multiple genes, these particles have become promising candidates for research into cancer therapeutics, including colorectal cancer. The role of miRNA expression in the development and diagnosis of colorectal cancer is thoroughly described, in addition to various operating algorithms in miRNA gene therapy [48].

A very interesting issue in the case of colorectal cancer is the importance of nutritional choices based on specific dietary components that can modulate miRNA expression, thereby preventing or even treating this type of cancer.

### 1.3. Effect of Diet on miRNAs in Colorectal Cancer

For thousands of years, people have been experimenting with diet and different combinations of ingredients to alleviate symptoms or even treat disease. To quote Hippocrates: “Your food should be medicine and your medicine should be food”. It is known that food ingredients from various sources, for example, fruits and berries, cruciferous vegetables, and soybeans, can directly affect enzyme activity or alter the expression of enzymes involved in epigenetic gene regulation [49]. Discovering the relationship of individual food components to specific miRNAs, one can explain why food can act as a medicine. In the following sections, we present selected dietary components that correlate with various miRNAs in the prevention, treatment, chemoprevention, metastasis, and prognosis of colorectal cancer.

#### 1.3.1. Grape Seed Extract

Various production processes result in the formation of so-called by-products. One such by-product is grape seed extract (GSE), which is formed during the production of grape juice and wine. Proanthocyanidins are the most important component of grape seed extract. These compounds belong to the flavonoids, and they consist of mixtures of di-tri- and oligo-catechins and epicatechins [50]. Common knowledge about the salutary antioxidant activity of GSE directed researchers’ interest toward the mechanisms of action of this group of compounds. In an experiment by the team of Derry et al., mice with colon tumors were fed GSE for a long time. The researchers conducted an experiment in which mice were treated with GSE for 18 and 28 weeks at doses of 0.25% and 0.5%. The obtained results show that miR-19a, miR-20a, and let-7a were increased while miR-196a and miR-205 were reduced. GSE inhibits NF-κB activation and causes a significant reduction in colon tumor size in a dose-dependent manner [51]. The same authors also conclude that the upregulation of miR-19a, NF-κB, and miR-20a targets the HIF-1α pathway and its downregulation targets vascular endothelial growth factor (VEGF) [51]. The metastasis process is closely related to the production of blood vessels (angiogenesis), which in turn is regulated by VEGF. In the same study, Derry et al. report that miR-205, which is downregulated by GSE, also targets VEGF and is also known to interact with both the MAPK and NOTCH pathways [51]. Thus, miR-135b targets APC, which is a regulator of β-catenin. In this way, miR-135b levels decrease, which results in degradation of β-catenin levels [51,52]. The NF-κB pathway, which is a key pathway of proinflammatory signaling, is related to tumor progression [53]. Therefore, lowering the activity of this pathway causes a decrease in inflammatory markers in CRC such as iNOS, COX-2, and VEGF [51]. Therefore, the regulation of many miRNAs influences the anti-inflammatory and antitumor activity of GSE, and the fact that the cited studies were conducted on mice fed with the preparation speaks in favor of the use of GSE, which suggests the ease of use of such substances.

#### 1.3.2. Resveratrol

After GSE, another valuable compound that can be obtained from grapes is resveratrol. Resveratrol is a polyphenol that is found mainly in the skin of grapes, raspberries, mulberries, and blueberries. It is well known that this group of compounds exerts pro-health effects mainly due to their antioxidant potential. Its activity on miRNAs in colorectal cancer was demonstrated in several experiments. Very interesting results were obtained by Tili et al. [54]. They treated SW480 colon cancer cells with 50 µM resveratrol for 14 h and found blocking of the expression of several oncogenic miRNAs, such as miR-21, which are induced in chronic inflammation. They report that the expression of the tumor suppressor miRNA, miR-663, is significantly higher in cancer cells in comparison to its expression in untreated cells [54]. Researchers also noticed that the use of resveratrol in the treatment of colon cancer cells led to a reduction in TGFβ1 and its downstream effector SMAD3, as explained by the action of miR-663 on TGFβ1 transcripts [54]. The target gene for miR-96 is KRAS, and this pathway is important in CRC as resveratrol has antitumor activity on it. In turn, the operation of KRAS is based on the triggering of signaling via Erk and Akt kinases, and thus enzymes related to multiplication and viability [55]. In their research, Saud et al. found that the action of resveratrol increased the levels of miR-96 in mice with colorectal cancer and this in turn led to a reduction in KRAS levels [56]. Two other targets of interest include the upregulation of miR-101b and miR-455, which in turn leads to decreased levels of IL-6 and TNF-α, which are proinflammatory proteins known to be colon cancer promoters [57,58]. In the work of Tili et al., another interesting function of resveratrol, namely its ability to upregulate miR-663, tsmiR, which targets TGFβ1 transcripts in SW480 cells, can be noticed [54]. Since TGFβ1 acts as a tumor promoter in late-stage tumorigenesis by increasing angiogenesis and metastasis, lowering the level of TGFβ1 leads to inhibition of cancer cell proliferation. Moreover, resveratrol increases the level of miR-34a in DLD-1 and SW480 cells [59]. In another experiment, Kumazaki et al. showed that resveratrol inhibited the growth of human colon cancer cells by upregulating miR-34a, which in turn lowered the levels of the E2F3 and Sirt1 genes [59]. These reports allow us to conclude that because resveratrol exhibits anti-inflammatory and anti-cancer properties, this fact can be associated with its antioxidant properties [60].

#### 1.3.3. Curcumin

Curcumin is a polyphenol extracted from the Curcuma longa plant in 1815, which receives interest from scientists for its biological effects. The popular name “Indian saffron” is the term used for this rhizome, from which the polyphenolic compound curcumin is obtained [61]. It is well known for its antioxidant, anti-inflammatory, antimicrobial, and antiviral properties. Among these activities of curcumin, the most interesting is its anti-cancer potential, which has been extensively researched. Curcumin, in addition to its typical anti-cancer effect, is related to the fact that it reduces inflammation, i.e., it was shown to induce ROS-dependent downregulation of miR-17-5p, miR-20a, and miR-27a. The above miRNAs target the zinc finger and BTB domain with the proteins ZBTB4 and ZBTB10 [62]. In the above-mentioned study, it was found that curcumin was responsible for increasing the level of reactive oxygen species (ROS) in the cell and, at the same time, inducing miR-17-5p, miR-20a, and miR-27a downregulation. Furthermore, under the influence of glutathione (GSH), which is an inhibitor of ROS, levels of miRNA expression stabilized, which indicates the role of ROS in the downregulation of miRNA-17-5p, miR-20a, and miR-27a [62]. Another study demonstrated an inhibitory effect of curcumin on the canonical Wnt/β-catenin pathway by downregulating miRNA-130a in SW480 CRC cells [63]. As is reported, this pathway is involved in the growth and proliferation of cells when it is active, and the accumulation of β-catenin is often a characteristic feature of neoplastic cells [64]. When SW480 cells are treated with curcumin, inhibition of their proliferation and decreased levels of β-catenin can be found. In their research, Dou et al. showed that when miR-130a is overexpressed in curcumin-treated SW480 cells, cell proliferation and β-catenin levels are restored, showing that miR-130a is responsible for these changes [63].

#### 1.3.4. Quercetin

Quercetin is a very interesting chemical in the context of anti-cancer activity. It is a flavonoid found in many plants and food products, such as green tea, apples, onions, and red wine, and is important in preventing tumor formation in the large intestine due to its anti-inflammatory effects and pro-apoptotic mechanisms. Interesting results on the activity of quercitin are described by Noratto et al. They report that the flavonols of the Yampon fraction (Ilex vomitoria) containing quercetin increased the expression of miR-146a by acting on colon cancer cells [65]. Based on this finding, it is possible to explain to some extent the anti-inflammatory effects of quercetin through the reduction in NF-κB levels by miR-146a. Moreover, in HT-29 colon cancer cells, quercetin–resveratrol combination treatment reduced the proteins SP1, SP3, and SP4, transcription factors known to be overexpressed in colon cancer. Furthemore, the synergistic action of resveratrol and quercetin induced ZBTB10 through downregulation of miR-27a [66]. This is a milestone in research into the potential synergistic effects of bioactive dietary compounds in modulating microRNAs in colon cancer.

#### 1.3.5. Vitamin D

In epidemiological studies, a low vitamin D diet or circulating calcidiol (25-hydroxyvitamin D3) levels are linked to an increased risk of colorectal cancer, with vitamin D playing a protective role in colorectal cancer [67,68]. Vitamin D (1.25 (OH) 2 D3 was also found to antagonize Wnt signaling in human colon cancer cells in several ways [69] and affect inflammatory pathways involved in cancer progressions such as COX-2 and NF-κB [70]. Alvarez-Diaz et al. identified miR-22 as a target of 1.25 (OH) 2 D3 in human colon cancer cells and showed that they potentially mediate, in part, its inhibitory effects on cell proliferation and migration [71]. In contrast, in another experiment, it was observed that the active form of vitamin D increases the level of miR-627 and contributes to its anti-tumor activity in colon cancer by targeting the histone demethylase JMJD1A [72]. Thus, 1.25 (OH) 2 D3 is responsible for the proliferation of cancer cells both in vitro and in vivo. Based on these observations, it is possible to conclude that this vitamin has at least a partial protective effect and can therefore prevent colorectal cancer.

#### 1.3.6. ω-3-Polyunsaturated Fatty Acids

Recent years have been a period of growing interest in omega-3 (ω-3) polyunsaturated fatty acids (PUFAs) due to their different roles in promoting health and reducing disease risk. ω-3 PUFAs include α-linolenic acid (ALA; 18: 3 ω-3), stearidonic acid (SDA; 18: 4 ω-3), eicosapentaenoic acid (EPA; 20: 5 ω-3), docosapentaenoic acid (DPA22: 5-3), and docosahexaenoic acid (DHA; 22: 6-3). The potential protective effect of omega-3 fatty acids against inflammatory diseases, including cancer, was described [73,74]. Interestingly, the effect of fish oil prevents the downregulation of several miRNAs in the rat colon 34 weeks after azoxymethane injection, which is expressed by miR-15b, miR-107, let-7d, miR-191 and miR-324-5p, and corresponds to a significant reduction of colon tumor formation [75]. The results of other experiments indicate that meals rich in fish oil and pectin ingredients increase levels of miR-19b, miR-26b, and miR-203, and this manifests after downregulation of IGF1, IGF2, and transcription factor 4 [76]. As Tu et al. report, ω-3 PUFAs are accepted as dietary supplements and even prescription drugs in the USA. According to this group of researchers, evidence from epidemiological, clinical and pre-clinical studies indicates a beneficial effect of ω-3 PUFA in the fight against CRC and its protective effect in weakening IBD in humans and animals [77].Very interesting observations are made by Moradi Sarabi et al. [78]. They report that n-3 PUFAs can affect cellular miR-126 DNA methylation and inhibit VEGF expression according to the cell type in colorectal cancer. The researchers also claim that DHA is more effective than EPA and LA in all cases, leading them to conclude that the potential clinical use of n-3 PUFAs as anti-angiogenic agents in CRC therapy could be a promising development.

#### 1.3.7. Sulforaphane

Sulforaphane, which is a derivative of isothiocyanate, is found in some cruciferous plants, including kale, cabbage, and broccoli sprouts. Slaby et al. describe the induction of change in the expression of miRNAs, for example, miR-23b andmiR-27b tsmiRs upregulation and downregulation of miR-155 oncomiR, in sulforaphane-treated normal epithelial colon cell lines NCM460 and NCM356 [79]. The process that leads to metastasis and chemoresistance in colorectal cancer is the epithelial–mesenchymal transition (EMT) [80]. EMT allows polarized epithelial cells to adopt a mesenchymal phenotype but is inhibited by miRNA-23b [81]. Another study suggests that miR-23b downregulate FZD7 and MAP3K1, promoting metastasis in HCT116 line cells [82]. A different team of researchers report that SOCS1 (suppressor of cytokine signaling 1) exerts a suppressor effect in a colon cancer line of cells via inhibition of EMT and the spread of tumor cells [83]. Moreover, the effect of miR-155, which is responsible for the upregulation of Akt connecting to the 3’-UTR of the catalytic subunit of protein phosphatase 2 alpha (PPP2CA), which is a known Akt suppressor, was described [84]. Therefore, treatment with sulforaphane reduce the expression of miR-155, which also lowers tumor formation by modulating the Akt cascade. It is therefore a substance that can act by changing the functioning of signaling cascades in inflammatory processes, EMT, and telomerase, thus exerting an anti-cancer effect [85].

#### 1.3.8. Dietary Fiber

The beneficial effect of fiber on the digestive tract is well known. In addition, there is increasing scientific evidence in the literature that eating fiber-containing foods has anti-cancer properties. For example, for each 10 g increase of fiber daily intake, a reduction in risk of colorectal cancer of 10% is seen [86]. The protective effect of a high-fiber diet against the development of colon cancer is explained by the increased production of butyrate, which is a product of fiber fermentation in the gastrointestinal tract. In the studies conducted on HT29 and HCT116 lines by the team of Humphreys et al., the effect of butyrate on reduction in the expression of the miR-17-92 oncogenic cluster, which in turn cause upregulation of genes such as *PTEN*, *Bcl-2L11*, and *CDKN1A*, was noted. In another in vivo experiment, animals were fed with food containing red meat supplemented with butylated starch with a large amount of amylose at a dose of 40 g/day. It was found that the level of miR-17-92 returned to the baseline state, and therefore miR-21 stabilized [87]. In the work conducted by the research team of Hu et al., a reduction effect of butyrate on C-myc and an increase in p57 expression in CRC cells was demonstrated. In addition, an inducing effect of butyrate on the expression of p21, which is an essential element in the regulation of the cell cycle through the downregulation of miR-106b, was noted [88]. As confirmed by the research conducted in recent years by the team of Ali et al. on colorectal cancer cells, there is a new network of linkages between RNA and butyrate, and the mutual ability of miRNAs to enhance the anticancer properties of butyrate. Researchers showed that silencing the central target gene, *EIF4G2*, significantly improved the anticancer effect of butyrate on CRC cells [89].

#### 1.3.9. Diet-Microbiota

A separate and very interesting thread in the research is the participation of microorganisms in miRNA modulation and colorectal cancer. Microbes are known to produce bioactive compounds such as short-chain fatty acids (SCFAs), choline metabolites, and lipids, which are very important in triggering the host epigenome locally in the gut, but also distal to the liver, heart, and central nervous system [90]. We define microbiome as the collective genomic content of all microbes living in a specific environment, and it includes at least 1000 different species of known bacteria and over 3 million genes involved in roles such as breakdown of sugars, vitamin synthesis, immunity modulation, and drug metabolism [91]. In this context, reports on urolithins are interesting. These are substances formed because of the metabolism of ellagitannins by intestinal bacteria. Urolithins were shown to have broad effects in vitro and in vivo, including antioxidant, anti-inflammatory, anti-estrogenic, and anti-proliferative effects [92,93,94]. Interesting results were obtained by González-Sarrías et al., who tested the effects of selected ellagitannin metabolites or a mixture of metabolites at concentrations of 100 mM for 48 h. They report that tested substances inhibited cell proliferation and induced cell cycle arrest and apoptosis in colon cancer cells. In this study, the González-Sarrías et al. team identified the induction of the cyclin-dependent kinase 1A inhibitor (p21) as a common target of urolitins and concluded that p21 induction is combined with downregulation of onco-miR-224 or upregulation of the tumor suppressor miR-215 [95]. In turn, a team of researchers led by Nuñez-Sánchez studied the effect of a daily dose of 900 mg of pomegranate extract for 5–35 days on miRNA expression in colon tissue compared to tumor tissue in preoperative CRC patients compared to 10 control CRC patients in a randomized, double-blind, controlled study. Ingestion of pomegranate extract reversed the upregulation of various miRNAs caused by surgery and slightly decreased the expression of selected miRNAs in tumor tissue compared to normal tissue. Nevertheless, no relationship was found between tissue urolithin levels and the observed changes in miRNA expression [96]. In the literature, there are reports of the anti-CRC effect of some probiotics, which is manifested, for example, in the elimination of carcinogens, the release of anti-cancer substances, the improvement of intestinal permeability, the function of tight junctions, and the activity of enzymes [97]. The team of Zununi Vahed et al. showed that strains of lactic acid bacteria *Leuconostoc mesenteroides* isolated from traditional dairy products co-cultured with HT-29 cells downregulated two oncomirs, miR-21 and miR-200b, responsible for promoting apoptosis of CRC cells. In this case, apoptosis was a consequence of the upregulation of mitogen-activated protein kinase 1 (MAPK1), Bcl-2-associated protein 4 (Bax), and caspase 3, and downregulation of Akt, NF-κB, and BclXL [98]. A very interesting study was conducted by Yuan et al. [99]. Researchers identified 76 different miRNAs compared to normal colon cells. They found a significant correlation of miRNAs with the multiplicity of bacteria in the colon cancer environment, including *Providencia*, *Akkermansia*, *Bacteroides*, *Porphyromonas*, *Roseburia*, *Peptostreptococcus*, and *Fusobacterium*, of which *Akkermansia* spp. was the only strain associated with miRNAs involved in the CRC pathway. In addition, researchers found that miRNAs associated with *Fusobacterium* spp. were involved in the glycan biosynthetic pathway, which is postulated to increase glycan production by CRC cells, as well as the recruitment of pathogenic bacteria that attach to cells via the Fap2 protein and the promotion of CRC progression [99]. Another thread of research in the field of the microbiome is miRNAs (xenomirs) derived from food, which are regulators of gene expression and affect the relationship between mammals and microorganisms. Dietary plant-derived miRNAs modulate the composition of the intestinal microflora and regulate intestinal permeability [100]. An example of such an effect can be exosome-like nanoparticles containing miRNAs, which can affect gene expression in the microbial environment of the gut, as described by Teng et al. One of the conclusions of this team of scientists is the finding that the metabolic products of plant-derived exosomes have an inhibitory effect on the growth of *E. coli*, *Bacteroides fragilis*, and *Listeria spp*. without affecting *L. rhamnosus*, and that another plant-derived miRNA, gma-miR-396e, promotes the growth of *L. rhamnosus* by inhibiting expression of LexA and also ath-miR-167a, a key regulator of SpaC expression [101]. Although the research results are promising, as in the case of the above-mentioned food ingredients, more detailed research is needed.

The effect of dietary components previously presented on miRNA in colon cancer is briefly shown in Table 1.

## 2. Conclusions

The modern human’s diet has a very strong effect on health due to its content of highly processed ingredients. Therefore, there are increasing numbers of studies, mainly epidemiological, that also show the other positive side of the action of various food ingredients. Hence, it is also possible to distinguish a preventive action against diseases of civilization, in addition to supporting and even treating diseases, in particular cancers. In the context of the research cited in this review, the focus is on Western eating patterns, which adversely affect health, especially due to the close relationship between obesity and cancer. Nevertheless, even in such a diet, there are some ingredients, such as those identified in this review including curcumin, resveratrol, and, at least, vitamin D, which have significant therapeutic potential in the treatment of colorectal cancer due to their ability to affect the expression of miRNAs. Colorectal cancer is especially related to the type of diet and different nutrients. Considering the cited studies, one can confidently conclude that these ingredients have a significant therapeutic potential as support for the main therapy. In this paper, we presented examples of very specific mechanisms by which various miRNAs are involved in the protection of cells against cancer, the process of inhibiting neoplasticity, metastasis, and even treatment options. A very interesting direction of research, which may become the main direction of research in the future, is the action of microRNAs of plant origin, which can be transferred to mammalian organisms through inter-kingdom regulation, which adjusts the appropriate target genes to their participation in the process of carcinogenesis [102]. Yet, it should still be borne in mind that the mechanism of plant-derived microRNAs in colorectal cancer is still unclear. Answering the question posed in the title of this review, in the light of the available research results, it can be said that yes, dietary actives can affect miRNAs and alter the course or prevent colorectal cancer. However, it should be remembered that in vitro studies on cell lines are still the only authoritative source of data in the field of miRNA regulation by dietary components. It would be beneficial and certainly most desirable if the experimental level could be transferred to clinical trials. Nevertheless, this is still a matter of the future.

In addition to the ingredients described in our review, many other potentially anti-cancer nutritional substances are waiting to be explored. In this system, it is miRNA that will become the main player and target of research teams.

## Figures and Tables

**Table 1:** The effect of dietary components on miRNA in colon cancer.

Dietary Component	miRNA Change	miRNA-Related Targets/Changes	References
Grape seed extract	Upregulated miR-19a, miR-20a, let-7a Downregulated miR-196a, miR-205	Inhibited NF-κB activation and caused a significant reduction in colon tumor size	Derry et al. [51]
Resveratrol	Upregulated miR-663 miR-101b miR-455 Downregulated miR-21	Reduction of chronic inflammation, Decrease inIL-6 and TNF-α levels, Suppression of tumor	Tili et al. [54] Altamemi et al. [57] Chung et al. [58]
Curcumin	Downregulated miR-17-5p, miR-20a, miR-27a miR-130a	Reduction in inflammation, Inhibition of cell growth and proliferation by Wnt/β-catenin pathway downregulation	Gandhy et al. [62] Dou et al. [63]
Quercetin	Upregulated miR-146a	Reduction in inflammation by decreasing of NF-κB level	Noratto et al. [65]
Vitamin D	Downregulated miR-22	Inhibitory effects on cell proliferation and migration,	Alvarez-Diaz et al. [71]
	Upregulated miR-627	Anti-tumor activity by targeting the histone demethylase JMJD1A	Padi et al. [72]
ω-3-Polyunsaturated Fatty Acids	Downregulated miR-15b, miR-107, let-7d, miR-191, miR-324-5p	Reduction in colon tumor formation	Davidson et al. [75]
	Upregulated miR-19b, miR-26b, miR-203	Downregulation of IGF1, IGF2, and transcription factor 4	Shah et al. [76]
Sulforaphane	Upregulated miR-23b, miR-27b tsmiRs	Inhibition of EMT,	Castilla et al. [81]
	Downregulated miR-155 oncomiR	Upregulation of Akt connecting to the 3′-UTR of the catalytic subunit of (PPP2CA)-Akt suppressor	Bakirtzi et al. [84]
Dietary fiber	Downregulated miR-17-92	Upregulation of genes such as *PTEN*, *Bcl-2L11*, and *CDKN1A*	Humphreys et al. [87]
	miR-106b	Increase in p57 expression, Reduction effect on C-myc in CRC cells, Induction of the expression of p21	Hu et al. [88]
Diet-microbiota	Downregulated miR-224 Upregulated miR-215 Downregulated miR-21, miR-200b	Cell proliferation inhibition, cell cycle arrest, apoptosis induction by p21 induction Apoptosis promotion in CRC cells By upregulation of MAPK1, Bax, caspase-3 and downregulation of Akt, NF-κB, Bcl-XL	González-Sarrías et al. [95] Zununi Vahed et al. [98]

## Data Availability

Not applicable.
